# Evaluation of polyhydroxyalkanoate (PHA) synthesis by *Pichia* sp. TSLS24 yeast isolated in Vietnam

**DOI:** 10.1038/s41598-023-28220-z

**Published:** 2023-02-23

**Authors:** Nguyen Thi Tam Thu, Le Huy Hoang, Pham Kien Cuong, Nguyen Viet-Linh, Tran Thi Huyen Nga, Dang Dinh Kim, Yoong Kit Leong, Le Thi Nhi-Cong

**Affiliations:** 1 Institute of New Technology, Academy of Military Science and Technology, Hanoi, 10072 Vietnam; 2 Institute of Biotechnology, Vietnam Academy of Science and Technology, 18 Hoang Quoc Viet, CauGiay, Hanoi, 10072 Vietnam; 3 Graduate University of Science and Technology, Vietnam Academy of Science and Technology, Hanoi, 10072 Vietnam; 4 University of Science, Vietnam National University-Hanoi, Hanoi, 11400 Vietnam; 5 Institute of Environmental Technology, Vietnam Academy of Science and Technology, Hanoi, 10072 Vietnam; 6 Department of Chemical and Materials Engineering, Tunghai University, Taichung, 407224 Taiwan

**Keywords:** Biotechnology, Microbiology, Environmental sciences

## Abstract

Following the rising concern on environmental issues caused by conventional fossil-based plastics and depleting crude oil resources, polyhydroxyalkanoates (PHAs) are of great interest by scientists and biodegradable polymer market due to their outstanding properties which include high biodegradability in various conditions and processing flexibility. Many polyhydroxyalkanoate-synthesizing microorganisms, including normal and halophilic bacteria, as well as algae, have been investigated for their performance in polyhydroxyalkanoate production. However, to the best of our knowledge, there is still limited studies on PHAs-producing marine yeast. In the present study, a halophilic yeast strain isolated from Spratly Island in Vietnam were investigated for its potential in polyhydroxyalkanoate biosynthesis by growing the yeast in Zobell marine agar medium (ZMA) containing Nile red dye. The strain was identified by 26S rDNA analysis as *Pichia kudriavzevii* TSLS24 and registered at Genbank database under code OL757724. The amount of polyhydroxyalkanoates synthesized was quantified by measuring the intracellular materials (predicted as poly(3-hydroxybutyrate) -PHB) by gravimetric method and subsequently confirmed by Fourier transform infrared (FTIR) spectroscopic and nuclear magnetic resonance (NMR) spectroscopic analyses. Under optimal growth conditions of 35 °C and pH 7 with supplementation of glucose and yeast extract at 20 and 10 gL^−1^, the isolated strain achieved poly(3-hydroxybutyrate) content and concentration of 43.4% and 1.8 gL^−1^ after 7 days of cultivation. The poly(3-hydroxybutyrate) produced demonstrated excellent biodegradability with degradation rate of 28% after 28 days of incubation in sea water.

## Introduction

Biodegradable plastics are plastic products which can be degraded by living organisms such as yeast, fungi, bacteria, actinomycetes and others under certain conditions when being released into natural environments. The demand for biodegradable plastics is currently surging owing to their promising capability in mitigating pollution issues caused by conventional petroleum plastics. Therefore, many studies on bioplastics, including polylactic acid (PLA) and polyhydroxyalkanoates (PHAs), have been reported. Among various types of bioplastics, PHAs can be biologically and synthesized and completely degraded by microorganisms even in saline condition such as in sea water^1^. Besides its biodegradability and compostable properties, PHAs have promising potential of industrial-scale production as it can be easily converted into different forms with desired characteristics following their large variability in structure^2^. They demonstrated promising potentials in medical applications and production of adhesives, films, moulded goods, paper coatings, packaging, non-woven fabrics and performance additives^3^.

In nature, PHAs are mainly accumulated inside microorganisms in the form of insoluble particles in cytoplasm. Following the nutrients imbalance due to surplus of carbon sources or deficiency of nutrient sources, such as nitrogen, phosphorus, sulphur, or even oxygen, PHAs with high molecular weight (containing 10^3^–10^4^ monomers, 0.2–0.5 µm in size and the number of particles is 5–13 particles/cell) would be formed by the intracellular enzymatic system through transference of carbon source^4^. These insoluble particles serve as the carbon storage and energy reserve of the cell, which does not influence the cellular osmotic pressure even when it is accumulated in high concentrations^1^. There have been many studies on various types of PHA-producing bacteria, including *Pseudomonas, Bacillus, Alcaligenes, Azotobacter, Burkholderia* as well as halophilic bacteria such as *Halomonas, Haloferix*^5^^,^^6^*.*

Yeasts are potential host for the production of PHA polymers as they can utilize inexpensive substrates as carbon sources and they can be easily genetic engineered^7^. Moreover, they also have good tolerance towards high concentrations of sugars and organic acids. The yeast *Saccharomyces cerevisiae*, *Yarrowia lipolytica*, and *Pichia pastoris* have been reported to be capable to synthesize short-chain-length (such as poly(hydroxybutyrate), PHB) and medium-chain-length PHAs from glucose^5,6,8–10^. In addition, there are also studies on genetically engineered yeast strains for the production of PHA, including *Y. lipolytica*^11,12^*, S. cerevisiae*^13–15^*, Rhodotorula minuta* RY4^11^, *Kebeckera* spp.^16^ and *P. pastoris*^17^, though the yields are unsatisfactory for large-scale production. Microbes can accumulate PHA up to 90% of their dry cell weight and enhance PHA production, and characteristics can be achieved by modifications in the types of substrates, feeding strategies, culture conditions and/or genetic manipulations^18^ However, there are not many studies on PHAs production by wild type yeast strains, especially halophilic yeasts^10^. Therefore, it is necessary to investigate the potential of halophilic yeast strains isolated from marine environment as hyperproducer of PHAs.

*Pichia* is a popular yeast genus which could be found in soil, fruit peels and other environments, which has the ability to protect fruits from damage and inhibit fungus development. As *Pichia* possess outstanding tolerance to high glucose concentration and is generally recognized as safe (GRAS), it has been applied in ethanol fermentation for beer and wine production. Furthermore, *Pichia* have promising competency in PHAs production and was reported to be able to accumulate high PHAs content up to 78.9% under optimal growth conditions^19^. The purpose of the present study is to investigate PHA accumulating yeast isolated from Spratly Island, Vietnam,identify the structure of the obtained PHA; optimize several influencing parameters for the yeast PHA production as well as demonstrate the degradability of biopolymer produced. The results may give a hint to produce biodegradable PHA in large scale to replace chemical plastic in near future in Vietnam.

## Materials and methods

### Sample collection

Sediment samples were collected near the livestock waste treatment area of Spratly Island, Vietnam at 8° 38′ 46.58″ N–111° 55′ 21.07″ E.

Chemicals and media for experiments were from Sigma and Merck Chemical Co., Germany, otherwise specifically indicated.

### Isolation, screening of PHA producer and observation the cells under fluorescence microscopy

#### Isolation and screening

One gram of sediment sample was added to 100 ml sterilized water and mixed before diluted to 10^−4^ or 10^−5^ times. Zobell marine agar medium (ZMA) (Hi-Media) was used to isolate halophilic yeasts. ZMA medium was prepared in sea water (90%, v/v) supplemented with 2 gL^−1^ of sucrose to enrich the growth of yeast and the dilution plates were incubated at 30 °C for 72 h. The distinct viable colonies were stained with 0.5 mgL^−1^ Nile red and observed after 48 h under ultraviolet light at wavelength of 235 nm. Colonies of pink or orange color were considered as PHA-producing yeast strains. Those strains were selected to be independently sub-cultured, and their glycerol stocks were prepared and preserved at 4 °C for further use^20^.

#### Observation of PHA producing cells under fluorescence microscopy

The selected strains were cultured in liquid ZMA medium supplemented with 10 gL^−1^ for 48 h. Then, the cultivation was supplemented with 1 ml of 0.5 mgL^−1^ Nile red and incubated at 37 °C for 1 h. The culture was centrifuged to remove the supernatant. The biomass pellet was smeared on slides and observed under Axioplus fluorescence microscope (Carl Zeiss, Germany) at magnification of 40 times, excitation wavelength of 550 nm and emission wavelength of 570 nm. The PHA granules accumulated in the cells were showing orange color^10^. The cellular morphology was also observed.

### Identification of the isolated yeast strain

The purified colony was used for total DNA extraction by a kit set (Chromous Biotech Pvt Ltd, Bangalore, India). The partial 26S rDNA was sequenced and Internal Transcribed Sequence (ITS) 1, 5.8S, ITS2 including Domain (D) 1, D2 regions of Large Subunit (LSU) were completed. The 26S rDNA sequences were multiplied using universal primers ITS1 (5′TCC GTA GGT GAA CCT GCG G-3′) and ITS4 (5′-TCC TCC GCT TAT TGA TAT GC-3′) by a PCR thermal cycler (Mycycler, Germany). The PCR product was subsequently purified by using clean up PCR SV kit (GeneAll ExpinTM, Korea). The DNA concentration was measured by nanodrop equipment (Colibri 75173, Germany) and sequences was analyzed by Sanger Sequencing machine (Germany). The 26S rDNA gene sequence was compared to related yeast sequences using NCBI Mega BLAST for their pairwise identification. The novel sequence was registered at GenBank (NCBI). CLUSTAL-W software was used to align the multiple alignments of these sequences. The neighbor-joining method was used to construct the phylogenetic tree using tree view software X^21^.

### Evaluation of saline tolerance of the isolates

The isolates were cultivated in 100 mL working volume of ZMA medium supplemented with different concentration of NaCl including 2, 3, 5, 7, 10 and 12% with similar initial inoculation concentration (OD_600 nm_ = 0.3). The cultivation was performed in a rotary incubator at 30 °C for 48 h. The growth of biomass was observed by measuring the optical density at wavelength of 600 nm at different periods of 0, 3, 6, 12, 18, 24, 30, 36 and 48 h after cultivation. Pure ZMA medium was used as the control.

### Extraction, recovery and quantification of PHA from selected yeast strain

#### Extraction

After finished cultivation, 50 ml of culture medium was centrifuged at 7000 rpm for 10 min at 4 °C to obtain biomass pellet. The supernatant was removed and the biomass pellet was washed twice with distilled water and dried in a hot air oven at 55 °C, then weighed on weighing scale to measure the dried weight of biomass (m_1_). The rest of culture medium was centrifuged for PHA extraction. The cell pellets were dissolved in 5% NaClO solution and incubated at 37 °C for 1 h. Then, 50 ml chloroform was added and the solution was incubated at 37 °C for another 1 h. Subsequently, the solution was centrifuged to remove the upper phase. The lower phase containing PHAs was then diluted in chloroform and supplemented with a similar volume of mixture ice-cold methanol (9:1 ratio) (v/v) for PHAs precipitation. Then, the solution containing precipitated PHAs was vortexed for 2 min and centrifuged at 10,000 rpm for 10 min. The supernatant was removed and the PHAs pellet was dried at 40 °C. The weight of purified PHA was measured (m_2_). The PHA content was calculated using the following formula:

$$\% PHA = \frac{{m_{2} }}{{m_{1} }} \times 100$$

#### Fourier transform infrared (FTIR) spectroscopic analysis

The functional groups present in the extracted PHA was evaluated using a Spectrum Two FT-IR spectrometer (PerkinElmer, England). Briefly, 1 mg PHA was diluted in 7 ml chloroform and 1 drop of the mixture was overlaid on FTIR KBr disk. The infrared spectra (IR) frequency was read in range of 400–4000 cm^−1^ at spectral resolution of 4 cm^−1^ in vacuum pressure^22^.

#### Nuclear magnetic resonance (NMR) spectroscopic analysis

The chemical structure of the extracted compound produced by the isolate was identified using proton NMR spectroscopic analysis. The sample was prepared by dissolving extracted PHA (6 mg) in 0.5 mL deuterated chloroform (CDCl_3_). The 1H NMR and 13C NMR spectrums were recorded at 125 and 400 MHz using a Bruker Advance III NMR spectrophotometer (Harwell, England). Probe temperature (25 °C) and tetramethylsilane as internal standard were used for this investigation^23^.

#### Gas chromatography—mass spectra (GC–MS) analysis

The analysis system consists of a GC 8000 gas chromatograph (Fisons Instruments, Mainz, Germany) equipped with a 30 m DB5 ms column (0.25 mm-by 0.33 μm film; J&W Scientific, USA) and a mass selective detector MD 800 (Fisons Instruments) operating at 70 eV or a TSQ 700 (Finnigan Corp., San Jose, Calif.) triple quadrupole mass spectrometer operated in a single quadrupole mode (Q1). A temperature programm from 60 to 290 °C (10 °C/min) was applied to separate the structure fragments on the column^24^.

#### Quantification of PHA

The purity of PHA was determined using the crotonic acid method^25,26^. In brief, 5 mg of PHA extracted mixture was added into 10 ml concentrated H_2_SO_4_ and then boiled at 100 °C for 30 min to convert PHA to crotonic acid. The standard calibration curve of crotonic acid was constructed by using pure PHA standard purchased from Sigma. The quantity of PHA in the extraction mixture was determined by comparing the amount of crotonic acid produced to those of the standard calibration curve. A negative control was also employed by using H_2_SO_4_ solution without PHA addition. The purity of PHA was calculated as the percentage of yeast-derived PHA in comparison to the calibration curve of the 5 mg PHA standard^27^.

### Screening of significant influencing factors on growth and PHA production of the marine yeast

Various influencing factors including temperature (15, 20, 25, 30, 35 and 40 °C), pH values (4, 5, 6, 7, 8 and 9), carbon sources (glucose, mannitose, lactose, starch, sucrose), nitrogen sources (peptone, yeast extract, ammonium sulfate, ammonium chloride, ammonium nitrate, urea) were optimized to obtain the optimal conditions for yeast growth and PHA production. Effects of nitrogen limitation on production of PHA by marine yeast using the screened carbon and nitrogen sources was also investigated. Different carbon/nitrogen (C:N, w/w) ratios in the range of 10:1, 20:1, 30:1, 40:1, 50:1, 60:1 and 80:1 were investigated to identify the optimal C:N ratio. All cultivation experiments were conducted in Erlenmeyer flasks of 250 mL with 100 mL operating volume for 72 h at 30 °C under rotary incubator. The maximum PHA content (%) and PHA concentration (gL^−1^) produced by the yeast strain were attained based on the total cell dry weight (gL^−1^). Furthermore, the yeast cultivation was performed at different operating volumes including 200, 500, 1000, 2000 and 5000 ml to obtain suitable volume for PHA production.

### Evaluation of biodegradability of PHA film formed by the selected strain in sea water

PHA film was formed by dissolving obtained PHA in chloroform supplemented with 4% PEG (to reduce the hardness of the film) and then the solution was poured into petri dishes of 9.8 mm in diameter. The petri dishes were left in the hood to allow evaporation of solvent to form PHA film. The PHA film with the thickness of 0.035 mm, area of 75.4 mm^2^, weight of 1 g/L was then incubated in medium containing 5 g/L glucose, 1 g/L yeast extract and sea water (3% NaCl) in flasks. Three groups were subjected to this experiment, including a control group using sterilized sea water, a group using natural sea water (to observe the bio-attenuation of natural microorganism consortium in sea water) and a group using sterilized sea water supplemented with the *Pichia* sp. itself with the initial inoculation concentration of 0.5%. Flasks of all 3 groups were incubated for a total of 28 days at 30 °C and 200 rpm to investigate the biodegradability of the PHA films. The PHA films were weighed gravimetrically at the beginning and after 28 days. The structure of films before and after decomposition was analyzed by employing scanning electron microscopy (SEM).

### Scanning electron microscopy (SEM) analysis

The SEM was used to observe the surface topological of the PHA films. Films was put onto sample holders (covered with carbon tapes), then placed into JEOL JSM6400 SEM system and analyzed following manufacturer’s manuals^22^.

### Statistical analysis

All experiments were conducted in triplicates (n = 3). Statistical analysis of the results was performed using the standard software package of Microsoft Excel. The results were represented as mean with standard deviation (mean ± SD). Significance between means tested using Student’s t test was of *p* < 0.05.

## Results and discussion

### Isolation and screening of PHA producer

**Figure 1:**
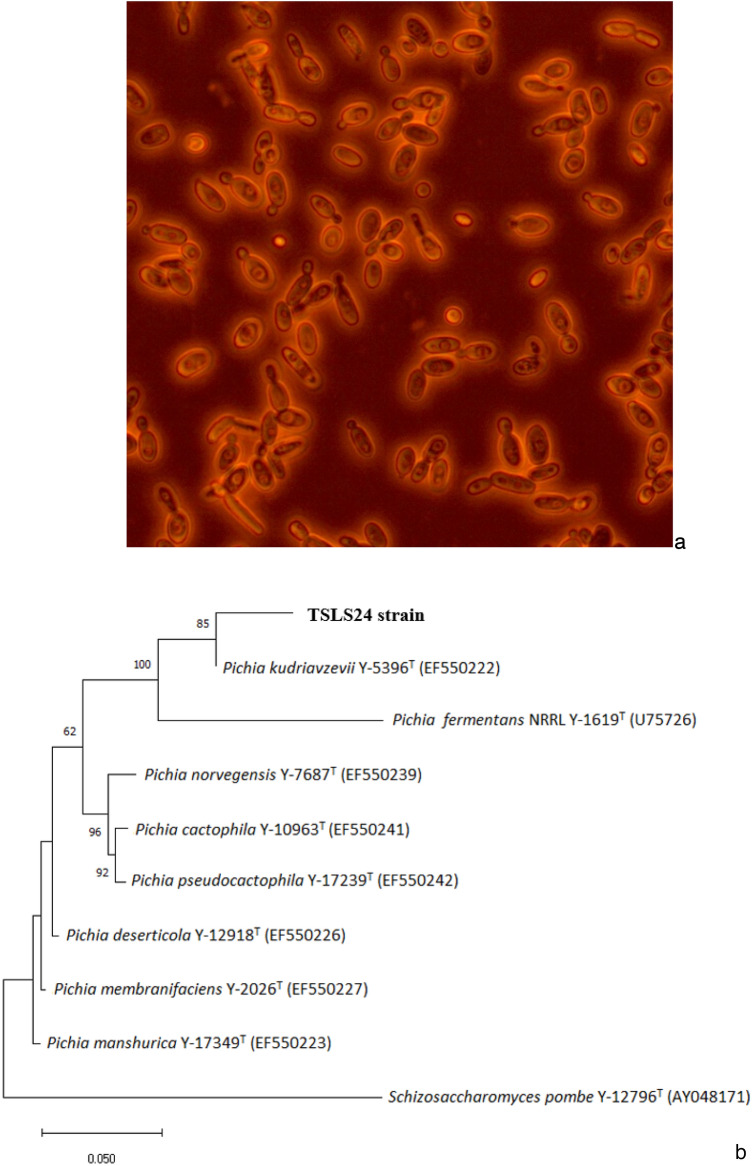
(**a**), PHA producing yeasts TSLS24 under fluorescence microscopy; (**b**), Phylogenetic tree constructed by the neighbor-joining method based on D1/D2 domain of 26S (LSU) rRNA gene sequences of close-related species, by MEGA-X software with neighbor-Joining analysis, indicates position of isolates G1-4(1) and G1-12(3). Bootstraps value (%) was based on 1,000 replications.

Microorganisms were considered as cell factories for the production of polyhydroxyalkanoates^28^. From collected sediment samples, a halophilic yeast strain named as TSLS24 was isolated using ZMA medium. By using Nile red staining and observing under fluorescence microscopy, the TSLS24 strain was orange fluorescence, then it could be a PHA producing organisms (Fig. 1a). The morphology of the yeast strain TSLS24 was ivory, colonies bearing an entire margin, circular shaped, convex, slightly rough surface with one colony size is approximately 2–3.5 mm in diameter. The strain does not excrete any pigment into the culture medium. The TSLS24 cells are long oval-shaped and it has one pole budding. The cell is approximately 4.0–10.0 × 2.5–5.5 µm in size.

### Identification of the PHA-producing yeast strain

26S rDNA gene sequence of TSLS24 related to those of the neighboring yeast sequences was performed by employing NCBI Mega BLAST for its pairwise identification. The phylogenetic analysis was evaluated based on partial sequence of 26S rDNA and complete sequence of ITS 1, 5.8S ribosomal RNA gene, ITS 2 and LSU ribosomal RNA gene of TSLS24. These results confirmed that TSLS24’s genome matches closest to the genus *Pichia kudriavzevii* with prominent uniqueness (100%), similar to a previously registered genus *Pichia kudriavzevii* Y-5396 T (GenBank: EF550222). The isolate was named as *Pichia kudriavzevii* TSLS24 and registered in GenBank with identity number of OL757724. CLUSTAL-W software was used to affiliate multiple alignments of these sequences and the phylogenetic tree was insinuated using the neighbor-joining method (Fig. 1b). Morphology of TSLS24 is analogous to that of *P. kudriavzevii* HOP-1, which was described as whitish to creamish in color with raised surface and oval, ellipsoidal to elongated-shaped size under SEM^29^. Several yeast species were affirmed to have the ability of producing PHA such as *Candida bombicola*^30^, *Candida tropicalis* BPU1^31^, etc. However, very few *Pichia* sp. was reported as a good PHA producer^19^. *Pichia kudriavzevii* VIT-NN02, isolated from the marine mangrove at 24 Parganas, Indian Sundarban, where the salinity increases up 30% throughout the entire year, was recognized as a potential PHA producer^19^. *Pichia kudriavzevii* was also found as bio-ethanol producing yeast which could use for fermentation at pilot-scale^32^).

### Saline tolerance of the isolate

To evaluate the saline tolerance of the TSLS24, a range of NaCl concentrations including 2, 3, 5, 7, 10 and 12% was established. According to Quillaguamán et al.^33^ and Kourmentza et al.^34^ the saline tolerant or halophilic strains could be used to obtain PHA by hypo-osmotic shock instead of using solvents. It is considered that halophilic microorganism could be used as biocatalysts for industrial PHA production^33,34^. The results showed that the cultivation of *Pichia* sp. TSLS24 in ZMA medium supplemented with 7% NaCl achieved the optimum biomass concentration of OD_600_ of 2.2 ± 0.2 after cultivation for 48 h (initial OD_600 nm_ = 0.3), equally to 1.15 ± 0.14 (g/L). On the other hand, culture with 10% NaCl gave OD_600_ of 1.5 ± 0.2, equal to 0.88 ± 0.06 (g/L). There is no growth of yeast inoculated in medium supplemented with 12% NaCl. Therefore, the isolate was determined as a halophilic yeast which can tolerate NaCl concentration up to 10%. Several halophilic bacterial strains were reported as PHA producers, including *Halomonas* sp.^35^, *Haloferax mediterranei*^8^, Alsafadi et al.^9^, *Halogeometricum borinquense*^36^, and others. However, studies on halophilic PHA-producing *P. kudriavzevii* strains are limited. The obtained results therefore provide more information on production of PHA from *Pichia*.

### Extraction, recovery and quantification of PHA from *P. kudriavzevii* TSLS24

#### FTIR analysis

**Figure 2:**
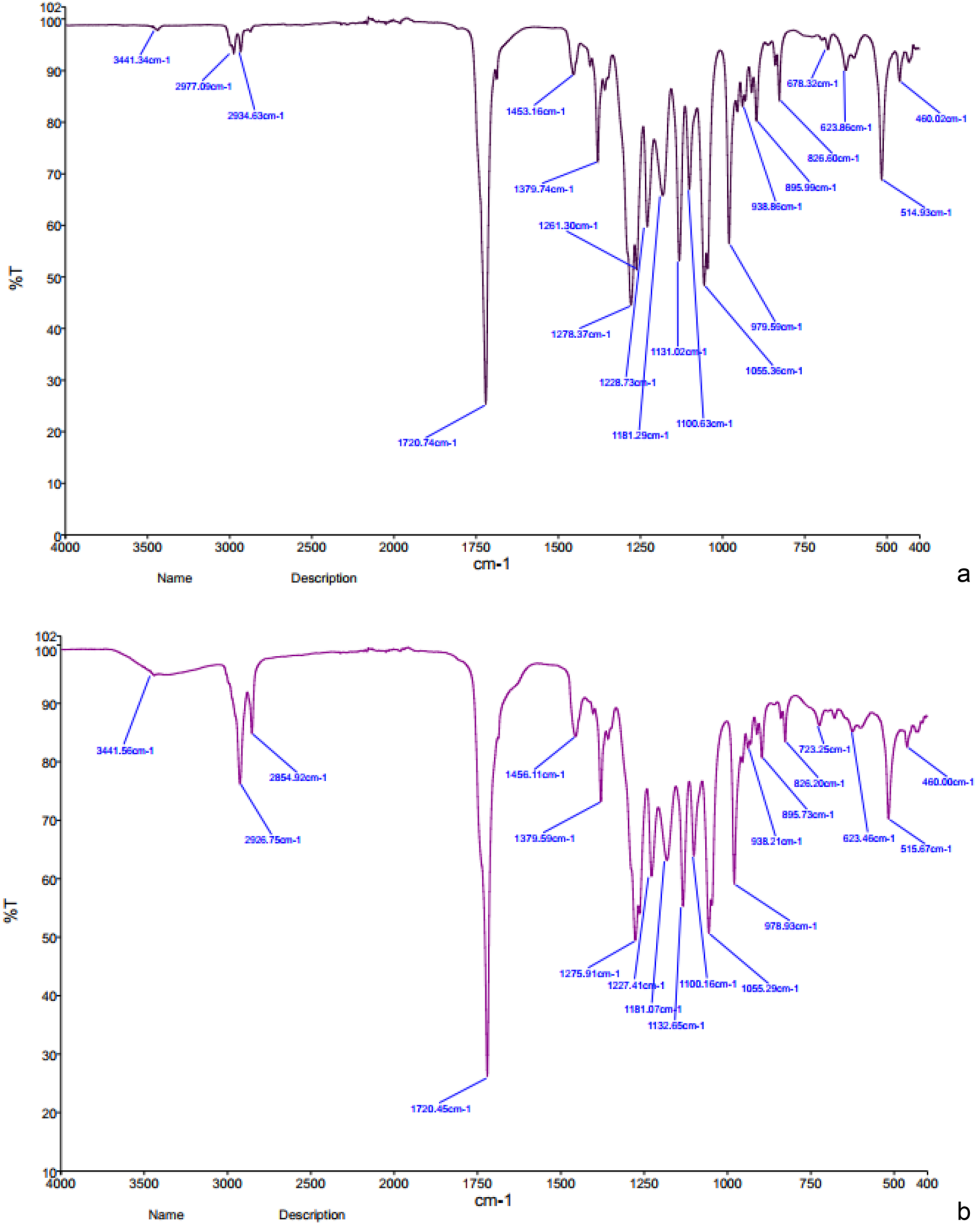

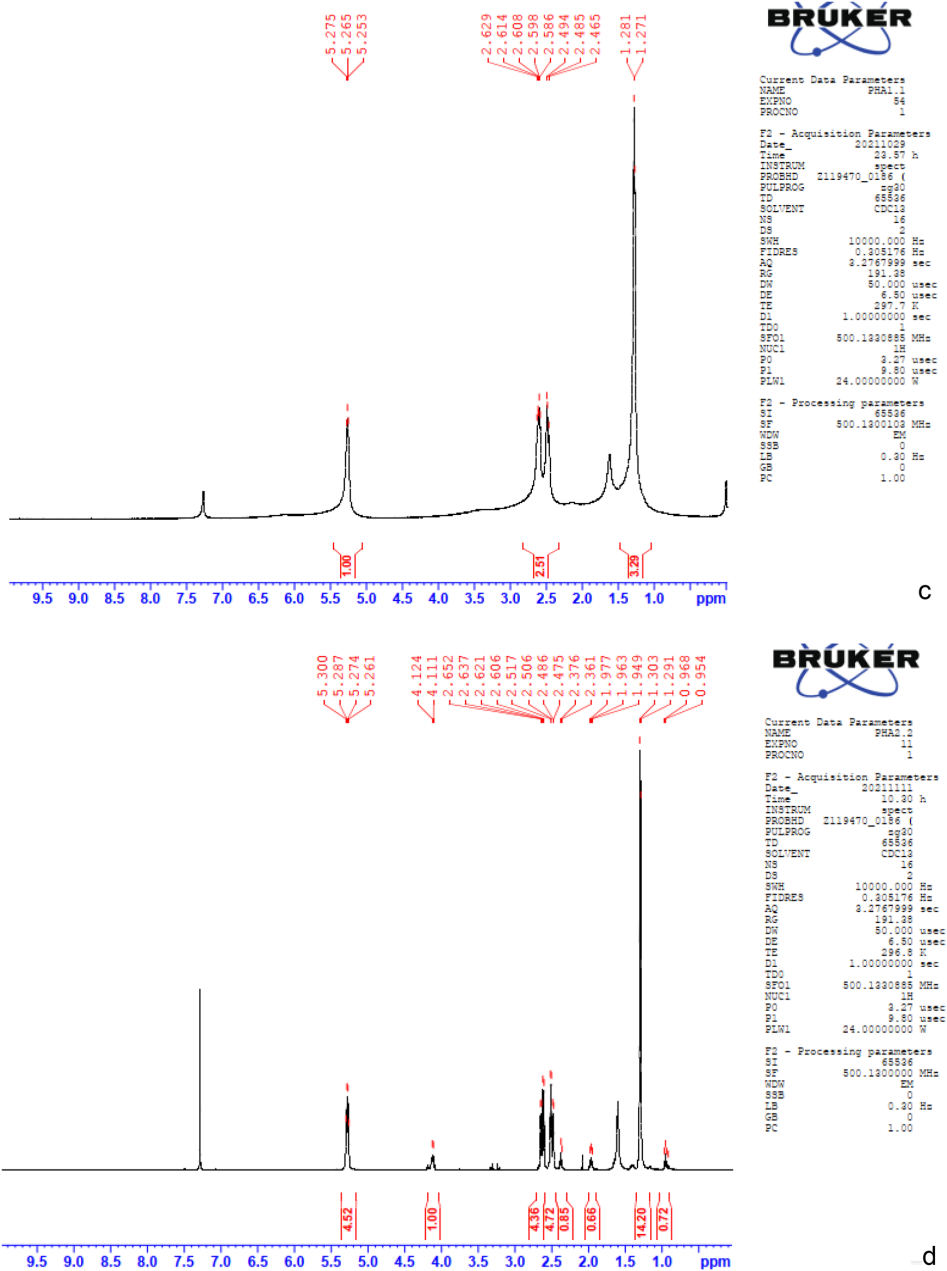

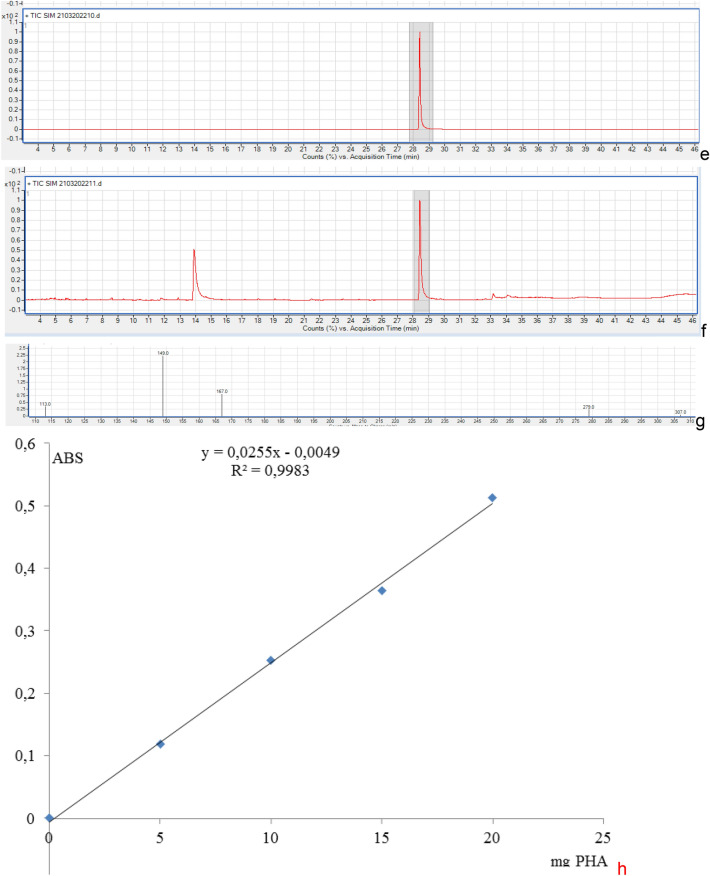
(**a**), FTIR spectrometer of PHA standard; (**b**), PHA produced by *Pichia* sp. TSLS24; (**c**), HNMR analysis of PHA standard (Sigma); (**d**) PHA produced by *Pichia* sp. TSLS24; (**e**), GC-MSn analysis of PHA standard (Sigma); (**f**) PHA produced by *Pichia* sp. TSLS24; (**g**), mass spectra of accumulated PHA by TSLS24; (**h**), Crotonic standard line for PHA measurement.

The PHA extracted from *Pichia* sp. TSLS24 was analyzed by FT-IR in comparison to PHA standard (Sigma). The spectra shown in Fig. 2a,b revealed that the absorption band appeared at 3441 cm^−1^ representing O–H bending. The broad nature and lower value of frequency indicated that the OH is free hydrogen-bonded. The prominent peaks at 2854 cm^−1^ and 2934 cm^−1^ showed influential –CH_2_ and –CH_3_ stretching groups of alkanes. The peak of 1720 cm^−1^ was the lengthening group C = O of ester group in the PHA. Group –CH(CH_2_)_2_– was presented at the peak of 1379 cm^−1^. Alkane (C–H) stretching bonds were observed at 1278 cm^−1^. Two strong C–O stretching bonds occurred at 1100 cm^−1^. A similar result was reported by Ojha and Das^19^, where FTIR analysis of PHAs from *Bacillus* such as *B. cereus*, *B. mycoides* and *B. thuringiensis* and from another *Pichia kudriavzevii* VIT-NN02 was exhibited.

#### NMR analysis

The monomeric structure of the extracted compound by *Pichia* sp. TSLS24 was revealed by 1H and 13C NMR spectrums as depicted in Fig. 2c,d. The signal at 1.2 ppm was related to the solvent, CDCl_3_^7^. The peaks existing at 2.4 and 2.5 ppm were corresponded to the methylene (–CH–(CH_2_)–CO–) group closest to an asymmetric carbon atom with 1 proton. The 1H NMR spectrum exhibited signals of the methane protons (–CH–) at 5.2 ppm signifying –O–(CH–) CH_2_– linking at carbon number 3. However, the extracted PHA has several tiny peaks at 1.9 and 4.1 ppm.

#### GC–MS analysis

According to GC–MS analysis, the accumulated PHA by TSLS24 has the similar retention time with the standard purchased PHA at 28.3 min (Fig. 2e,f). In the Fig. 2g, the mass spectra indicated a base peak ion at *m/z* 279 with fragment ions at *m/z* 167 [–O–CH(CH_3_)–CH_2_C(O)–O–CH(CH_3_)–CH_2_C(O)]; 149 [–O–CH(CH_3_)–CH_2_C(O)–O–CH(CH_3_)–CH_2_C–] and 113 [–O–CH(CH_3_)–CH_2_C(O)–O–C–]. In fact, the fragment [–O–CH(CH_3_)–CH_2_C(O)–O–CH(CH_3_)–CH_2_C(O)] was 172; [–O–CH(CH_3_)–CH_2_C(O)–O–CH(CH_3_)–CH_2_C–] was 156 and [–O–CH(CH_3_)–CH_2_C(O)–O–C–] was 114 but in the chromatography has only the *m/z* of 167, 149 and 113. It could be explained that during the analysis, several ion H^+^ in methyl groups were disappeared. The type of PHA produced by *P. kudriavzevii*. TSLS24 by FT-IR, NMR and GC analyses was shown to be homopolymer of standard poly(3-hydroxybutyrate) (PHB).

#### Quantification of PHA from P. kudriavzevii TSLS24

According to Law and Slepecky^25^ and Lee et al.^26^, PHA was supplemented by concentrated H_2_SO_4_ and then boiled at 100 °C for 30 min to convert PHA to crotonic acid. Therefore in this study, commercial PHA was used as internal standard/positive control and the crotonic acid standard line was constructed. According to the crotonic acid standard line (Fig. 2h), the concentration of PHA produced by *P. kudriavzevii* TSLS24 was estimated as 4.57 ± 0.35 mg out of 5.0 mg sample (w/w). This result indicated that the purity of the extracted PHA was 91.38% in comparison to the standard.

### Screening of influencing factors affecting cell growth and PHA production

The production of PHA in microbes is a complex process, which is affected by nutritional and environmental factors, including temperature, pH, carbon source, nitrogen source and C/N ratio. Therefore, there is a need to optimize these influencing parameters to maximize PHA production from the isolated strain.

#### Temperature

The *Pichia* sp. TSLS24 was cultivated in ZMA medium at different temperature for 96 h. Table 1 showed that TSLS24 could grow well and produced PHA in a wide range of temperature from 15 to 45 °C. However, at higher end of temperature range of 40–45 °C, the PHA content only reached 24.3–26.2% of dried biomass; the biomass concentrations were 2.84 and 2.18 gL^−1^ and the PHA concentrations were 0.74 and 0.52 gL^−1^, respectively. The lower temperature at 15 °C, the biomass and PHA concentrations were 2.55 and 0.76 gL^−1^. More optimum temperature range for PHA production are from 20 to 35 °C where the PHA content and concentration were more than 40% and 1.12 gL^−1^. With the most optimum cultivation temperature ranging from 30 to 35 °C, the obtained biomass concentration was more than 3 gL^−1^ with PHA content of approximately 47%. The PHA content and concentration reached a maximum of 47.5% and 1.65 gL^−1^ under optimal cultivation temperature of 35 °C. These results were in agreement with other PHA-producing yeast strains Ojha and Das^19^. Besides, the wide range of viable temperature, especially the higher end of 40–45 °C, as well as the optimal temperature range were in accordance with published studies on productivity of other compounds in yeast, such as the heat-tolerant *Pichia kudriavzevii* HOP-1, which was reported to be able to produce ethanol with good efficiency at 40–45 °C^29^^,^^37^. As a result, cultivation temperature of 35 °C was selected for subsequent experiment.

**Table 1:** Effect of temperature on the growth and PHA synthesis of *Pichia* sp. TSLS24.

Temperature (°C)	Biomass concentration (g/L)	PHA concentration (g/L)	PHA content (%)
15	2.55 ± 0.13^a^	0.76 ± 0.14^a^	30.1 ± 6.6^a^
20	2.78 ± 0.15^ab^	1.12 ± 0.13^b^	40.4 ± 5.5^ab^
25	2.97 ± 0.22^b^	1.27 ± 0.08^b^	43.1 ± 4.5^b^
30	3.15 ± 0.13^b^	1.48 ± 0.06^c^	47.0 ± 3.0^b^
35	3.48 ± 0.08^c^	1.65 ± 0.12^c^	47.5 ± 2.8^b^
40	2.84 ± 0.11^ab^	0.74 ± 0.05^a^	26.2 ± 2.3^a^
45	2.18 ± 0.09^d^	0.52 ± 0.13^a^	24.3 ± 6.7^a^

In a column, data with different superscript are significant with *P* < 0.05.

#### pH

The experiments were conducted with initial medium pH of 4, 5, 6, 7, 8 and 9 in a similar manner to those with temperature with results were presented in Table 2. The obtained biomass showed that TSLS24 could grow in all pH values. The biomass concentrations of the cultivations with the pH 4 and 5 were 0.98 and 1.26 gL^−1^; the PHA concentrations were 0.12 and 0.35 gL^−1^; equally to 12.2 and 27.8% of the PHA contents, respectively. With the range of pH 6–9, PHA accumulation in *Pichia* sp. TSLS24 was high and the optimal value for PHA production is pH 7. At pH 7, biomass production was the highest (2.78 ± 0.17 gL^−1^) and the PHA content reached the maximum of 46.5%, which allowed the PHA concentration to reach the highest of 1.28 ± 0.20 gL^−1^. For slightly alkaline pH value of 8 and 9, the PHA content maintained at the range of 45.8–46.4%, though the biomass concentration started to decline following the rise of pH values, in details biomass concentrations were 2.24 and 1.84 gL^−1^ and PHA concentrations were 1.02 and 0.84 gL^−1^.

**Table 2:** Effect of pH on the growth and PHA synthesis of *Pichia* sp. TSLS24.

pH	Biomass concentration (g/L)	PHA concentration (g/L)	PHA content (%)
4	0.98 ± 0.18^a^	0.12 ± 0.05^a^	12.2 ± 3.4^a^
5	1.26 ± 0.20^a^	0.35 ± 0.06^b^	27.8 ± 0.8^b^
6	2.35 ± 0.11^b^	0.99 ± 0.15^c^	41.9 ± 5.8^c^
7	2.78 ± 0.17^c^	1.28 ± 0.20^c^	46.5 ± 8.7^c^
8	2.24 ± 0.13^b^	1.02 ± 0.25^c^	45.8 ± 12.3^c^
9	1.84 ± 0.24^d^	0.84 ± 0.14^c^	46.4 ± 9.9^c^

In a column, data with different superscript are significant with P < 0.05.

According to other literatures, *Pichia* sp. was normally used in wine and beer fermentation with pH 3–4^29^. Besides, *Pichia* sp. is a valuable yeast genus for creating flavor and aroma for cocoa beans at pH 4^29^. In PHA production research, it was demonstrated that PHA production was significantly reduced at lower acidic pH compared to higher alkaline pH, where it was stable^19,38^. Our observation in the present study showed that *Pichia* sp. TSLS24 might also grow well in higher pH ranges from 6 to 9 and have high productivity of PHA and the optimal pH was 7. Similar results have been published for the *Alcaligenes latus* which attained PHB production of 10.30 ± 1.01 gL^−1^ with the optimal pH ranges from 6.0 to 7.5^39^. According to the optimization result, pH 7 was deployed for the subsequent experiments.

#### Carbon sources

Microbes can utilize different carbon sources for their growth and synthesis of PHA precursors. These precursors could be further polymerized into PHA depending on the metabolic pathways. Microbes such as *Pseudomonas* sp., *Halomonas* sp, and *Cupravidus* sp. were reported to be the best strains for PHA production in using six-carbon sugars e.g. glucose, cellulose, fructose, mannose, galactose, and maltose based on the glycolysis pathway^5,6^. Lactose was also reported to be utilized in the production of both PHA and biohydrogen by a two-step fermentation of deproteinized cheese whey, in which lactose is one of the major constituents^40^. Five-carbon sugars can also be utilized through the pentose phosphate pathway, however, there is only few microbes that could efficiently convert such sugars into PHA due to the fact that most PHA-producing strains which preferred 6-carbon sugars has low 5-carbon sugar metabolic activities^41^. In a rare case, *Burkholderia sacchari* DSM 17165 was reported to be able to effectively produce PHA with xylose and arabinose as carbon sources^41^. Therefore, in the present investigation, 20 gL^−1^ of six-carbon sugars, including glucose, mannitose, lactose, starch, or sucrose were employed as carbon source in ZMA medium with cultivation pH and temperature of pH 7 and 35 °C.

Table 3 demonstrated that all investigated carbon sources are suitable for yeast’s growth and PHA production. Among them, glucose is the best carbon source for PHA production with PHA concentration and content of 1.43 ± 0.07 gL^−1^ and 50.5%. The other carbon sources such as lactose, sucrose, starch and mannitose brought about the amount of biomass at 2.66, 2.42, 2.19 and 2.55 gL^−1^; and PHA at 1.26, 1.08, 0.97 and 0.99 gL^−1^, respectively. The flexibility of TSLS24 in utilizing different carbon sources is in accordance to previous studies. *P. kudriavzevii* was reported as a yeast which could flexibly utilize various carbon sources, such as glucose, sucrose, galactose, fructose, and mannose^29^. Moreover, the marine yeast *P. kudriavzevii* VIT-NN02 was reported to be able to use waste banana peels efficiently as carbon source for PHA production and achieved PHA content of 79%^19^. Furthermore, cheap carbon sources including sugarcane molasses, palm oil, and corn steep liquor were used by the yeast *Wickerhamomyces anomalus* VIT-NN01 isolated from sugarcane juice to produce PHA with PHA yield of 19.05 ± 0.3 gL^−1^ obtained after 96 h of cultivation^38^.

**Table 3:** Effect of carbon sources on the growth and PHA synthesis of *Pichia* sp. TSLS24.

Carbon source	Biomass concentration (g/L)	PHA concentration (g/L)	PHA content (%)
Glucose	2.84 ± 0.19^a^	1.43 ± 0.07^a^	50.5 ± 3.7^a^
Lactose	2.66 ± 0.15^ab^	1.26 ± 0.04^b^	47.5 ± 1.5^a^
Sucrose	2.42 ± 0.15^bc^	1.08 ± 0.09^c^	44.6 ± 4.8^ab^
Starch	2.19 ± 0.24^bc^	0.97 ± 0.07^c^	44.6 ± 3.7^ab^
Mannitose	2.55 ± 0.23^abc^	0.994 ± 0.09^c^	38.7 ± 3.2^b^

In a column, data with different superscript are significant with P < 0.05.

#### Nitrogen sources

In general, nitrogen is a crucial nutrient for growth of any microbes. PHA synthesis in microbes is sensitive to nitrogen, and most PHA-producing strains have different nitrogen source preferences. such as ammonia, urea, and nitrate^42^. Therefore, selection of nitrogen sources and concentrations is very important for microorganism growth and PHA production. In the present study, the cultivation of *P. kudriavzevii* TSLS24 was examined with different nitrogen sources including both organic and inorganic sources (Table 4).

**Table 4:** Effect of nitrogen sources on the growth and PHA synthesis of *Pichia* sp. TSLS24.

Nitrogen source	Biomass concentration (g/L)	PHA concentration (g/L)	PHA content (%)
Peptone	2.66 ± 0.16^a^	1.45 ± 0.06^a^	54.6 ± 3.6^a^
Yeast extract	2.70 ± 0.19^a^	1.49 ± 0.04^a^	55.2 ± 4.5^a^
(NH_4_)_2_SO_4_	1.42 ± 0.03^b^	0.45 ± 0.06^b^	31.6 ± 4.6^b^
NH_4_Cl	1.42 ± 0.03^b^	0.35 ± 0.05^b^	24.9 ± 3.7^bc^
NH_4_NO_3_	1.39 ± 0.02^b^	0.36 ± 0.03^b^	26.1 ± 2.5^b^
Urea	0.24 ± 0.04^c^	1.32 ± 0.03^c^	18.5 ± 2.9^c^

In a column, data with different superscript are significant with P < 0.05.

Among the nitrogen sources screened, yeast extract was shown to be the most suitable nitrogen source for the production of PHA by *P. kudriavzevii* TSLS24 (PHA content of 55.2%), followed by peptone (54.6%), ammonium sulfate (NH_4_)_2_SO_4_ (31.6%), ammonium nitrate (NH_4_NO_3_) (26.1%), ammonium chloride (NH_4_Cl) (24.9%), and urea (18.5%). Based on the findings, cultivation of TSLS24 with organic sources such as peptone and yeast extract gave higher biomass production (2.66–2.70 gL^−1^) and PHA content (54.6–55.2%) compared with inorganic nitrogen sources (0.24–1.42 gL^−1^ and 18.5–31.6%). Organic nitrogen sources have been also employed for cultivation of *Pichia* sp. As reported by Ojha and Das^19,38^, corn steep liquor and chicken feather hydrolysate has been utilized as cheap nitrogen source for cultivation of *Wickerhamomyces anomalus* VIT-NN01 and *Pichia kudriavzevii* VIT-NN02, respectively for production of PHA. These results suggested it could be interesting to examine the PHA production from *Pichia* sp. TSLS24 utilizing inexpensive organic nitrogen sources such as corn steep liquor, wheat bran, chicken feather and others.

#### C:N ratio

The ratio of C:N was demonstrated to affect yeast’s growth and PHA production^19^. The reason is that when carbon source in the cultivation medium is in excess whereas one of the other nutritional factors e.g. N, S, P is limited, yeast would be stimulated to produce PHA in the form of carbon storage granules inside the cell. In a nitrogen-deficient condition, microbes tend to reduce microbial protein production activities, along with increased PHA synthesis to be stored as a major reserve energy source^43^^,^^44^. Therefore, in this research, the effect of C:N ratio on PHA production by the *P. kudriavzevii* TSLS24 were evaluated with glucose and yeast extract were used as carbon and nitrogen sources. The concentration of glucose was fixed but the yeast extract concentrations were changed.

Table 5 demonstrated that 20:1 was the most effective C:N ratio for cell growth and PHA production, reaching PHA content and concentration of 50.9% and 1.53 ± 0.11 gL^−1^. However, there is no significant difference amongst all examined ratios. The results in the present study were in accordance to those by Wang et al.^44^, in which a reduction of PHA content in biomass was reported under nitrogen-rich conditions. In general, preference of C/N ratio varies with the type of strain, carbon, and nitrogen source. Different optimum C/N ratios were reported for PHA production in various microbes, such as 40:1 for *Pseudomonas mendocina*^45^, 21:1 for *Alcaligenes denitrifcans* A41^46^, and 15:1 for *Bacillus megaterium*^47^. C/N ratio of 20:1 was also reported to be optimum for the accumulation of poly-3-hydroxybutyrate-*co*-3-hydroxyhexanoate up to 67 wt% in *Ralstonia eutropha* using coffee waste oil as carbon source and ammonium chloride as nitrogen source^48^. In *Haloferax mediterranei*, it was reported that C/N ratio could affect the ability of simultaneous production of PHA and exopolysaccharides (EPS) simultaneous production where adjusting C/N ratio affect the proportion of synthesized PHA and EPS. At higher C/N ratio of 30, PHA was the main product, while at lower C/N ratio of 10, EPS was mainly produced^38^. The C/N ratio of 20:1 was selected for subsequent experiment (Supplementary file 1).

**Table 5:** Effect of C/N ratio on the growth and PHA synthesis of *Pichia* sp. TSLS24.

C/N Ratio	Biomass concentration (g/L)	PHA concentration (g/L)	PHA content (%)
10:01	2.82 ± 0.17^a^	1.31 ± 0.07^a^	46.6 ± 2.6^a^
20:1	3.01 ± 0.23^a^	1.53 ± 0.11^b^	50.9 ± 2.0^b^
30:1	2.56 ± 0.11^ab^	1.12 ± 0.13^ac^	43.9 ± 6.4^ac^
40:1	2.32 ± 0.14^b^	1.00 ± 0.13^c^	43.0 ± 4.6^c^
50:1	2.70 ± 0.16^ab^	1.34 ± 0.23^abc^	49.7 ± 8.6^abc^
60:1	2.77 ± 0.13^a^	1.12 ± 0.24^abc^	40.6 ± 8.7^ac^
80:1	2.66 ± 0.16^a^	1.13 ± 0.18^abc^	42.9 ± 8.2^ac^

#### Fermentation to produce PHA

*Pichia kudriavzevii* TSLS24 was cultivated under optimal conditions of pH 7, 35 °C with 20 g/L glucose and 1 g/L yeast extract. Fermentation was conducted with different operating volumes of 200, 500, 1000, 2000 and 5000 ml for 10 days. After every 3 h of cultivation, 10 gL^−1^ of glucose was supplemented to provide redundancy of glucose and ensure nitrogen limitation which stimulated the strain to produce PHA.

From Table 6, it can be observed that PHA content and concentration maintained in the range of 46.3–52.9% and 1.72–1.84 gL^−1^ regardless of fermentation volume. When the fermentation volume reached 5000 mL, PHA production reached 1.79 gL^−1^ with biomass concentration of 3.42 gL^−1^, achieving PHA content up to 52.9%. Therefore, *P. kudriavzevii* TSLS24 might be more suitable for larger-scale fermentation e.g. pilot-scale than laboratory-scale.

**Table 6:** Biomass and PHA production of *Pichia* sp. TSLS24 in different fermentation scales.

Fermentation Volume (mL)	Biomass concentration (g/L)	PHA concentration (g/L)	PHA content (%)
200	3.98 ± 0.38^a^	1.84 ± 0.16^a^	46.3 ± 1.4^a^
500	3.72 ± 0.19^a^	1.73 ± 0.10^a^	46.6 ± 3.9^a^
1000	3.95 ± 0.3^a^	1.72 ± 0.09^a^	43.9 ± 3.9^a^
2000	3.63 ± 0.19^a^	1.75 ± 0.09^a^	48.2 ± 1.6^a^
5000	3.42 ± 0.42^a^	1.79 ± 0.05^a^	52.9 ± 5.9^a^

Recently, there are a number of reports on PHA-producing microorganisms such as *Bacillus, Clostridium, Mycobacterium, Streptomyces, Fusobacterium, Burkholderia, Pseudomonas, Bordetella, Neisseria, Agrobacterium, Rhizobium, Ralstonia, Vibrio, Legionella, Acinetobacter, Sphingomonas, Rickettsia*^48–50^. The genetically engineered yeast strains such as *Kloeckera* sp.*, S. cerevisiae, Y. lipolytica, P. pastoris* and *Rhodotorula minuta* strain RY4 were also reported as PHA producers^11,13,15,16^. In 2018, Ojha and Das explored a potential yeast strain *Wickerhamomyces anomalus* isolated from natural sources. Though information on halophilic PHA producing yeast is limited meanwhile yeast is a potential option for the PHA production at pilot scale^28^.

### Evaluation of biodegradability of PHA film formed by *Pichia kudriavzevii* TSLS24 in sea water

**Figure 3:**
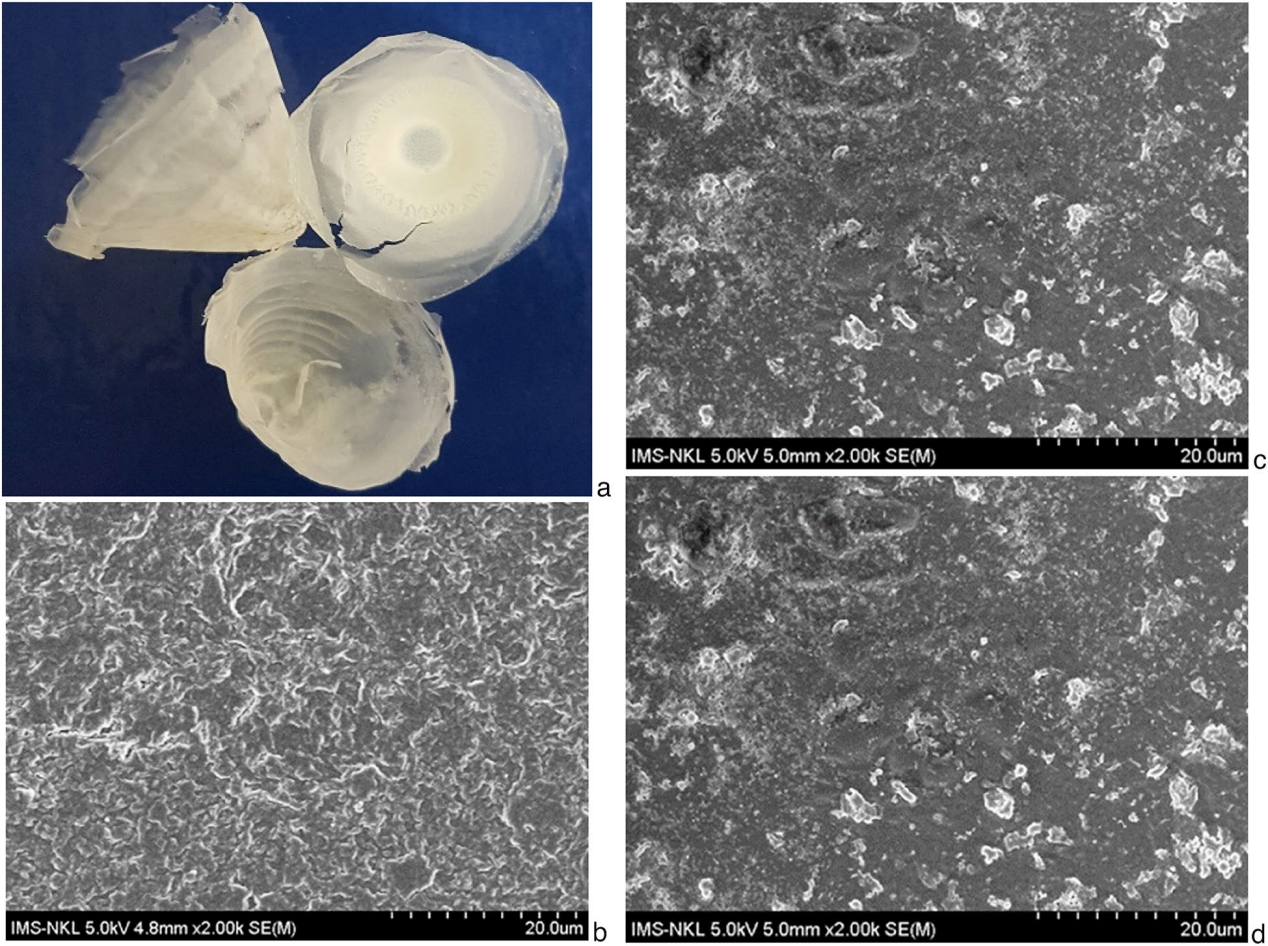
(**a**), PHA sample produced by *Pichia* sp. TSLS24; Changes in structure of PHA membranes after 28 days under TEM, magnification of 2000X: (**b**) Intact control PHA sample incubated in sterile sea water without microbes; (**c**) PHA sample degraded by natural microbes in non-sterile sea water; (**d**) PHA sample degraded by *Pichia* sp. TSLS24 in sterilized sea water.

Biodegradation study is crucial in order to understand the degradation rate of plastic materials and mechanisms involved. The results of degradation study showed that after 28 days, the weight of PHA film in control group (without microorganisms) did not change, meanwhile the weight was reduced by 28% and 19.5% in the experimental group inoculated with TSLS24 and the group using natural unpurified marine water. The structures of PHA films were observed and analyzed under SEM (Fig. 3). The structures of PHA film inoculated with TSLS24 experienced significant changes where the linkages among molecules were broken down by the TSLS24 (Fig. 3d), meanwhile the structure of the control without microbe was stable (Fig. 3b) and the structure of PHA film supplemented with natural sea water was slightly disrupted (Fig. 3c). The extracted PHA film was also degraded in marine water as the bio-attenuation. The results suggested that PHA produced by the strain TSLS24 could be easily degraded in saline environment (sea water) by the natural microbes exist in the environment. Compare to the degradation in soil and compost, the degradation of plastics in seawater is more complex due to low temperature, high salinity, high pressure, currents, and low nutrient levels (e.g., nitrate) of the marine environment, not to mention the variation between different seasons, different regions, or depth/pressure in the vertical direction affecting at least the water temperature. Therefore, in-depth investigations and evaluations is required to provide a crucial basis before any further practical application.

Since 1992, the degradation of PHAs in seawater have been reported in some seminal studies^44,51^. Degradation mechanism for PHAs in seawater followed the surface erosion, as it was also reported in soil and compost. However, the degradation rate of PHAs in seawater was significantly slower. The average degradation rate of PHA in the ocean was determined as 0.04–0.09 mg day^−1^ cm^−2^ where the thickness and geometric form of the product could affect the required time for degradation. For plastic bags of 0.035 mm thickness, the time for complete degradation was reported to be ranging from 25 days to 2 months, whereas a plastic bottle of 0.8 mm thickness could only be fully degraded after at least 1.5 years^1^.

## Conclusion

In the present investigation, a halophilic yeast strain *Pichia* sp. TSLS24 isolated from Spratly island in Vietnam showed its capability to grow and produce PHA efficiently in a wide range of temperature from 15 to 45 °C, wide pH range of pH 6–9, flexible carbon sources such as glucose, mannitose, lactose, starch and sucrose as well as different nitrogen sources. The efficiency of PHA production at different fermentation volumes was stable and achieved high PHA content of 52%. The extracted PHA was homologous with high purity (91.4%) and it was confirmed as purchased PHB by using FTIR, NMR and GC–MS analyses. and the accumulated PHA can be easily biodegraded by natural organisms, even in extreme conditions like in sea water. The future works include optimization of PHA extraction and production of PHA at pilot scale or in vivo. The results provide insights on PHA production by *P. kudriavzevii* TSLS24. Especially, the TSLS24 could be used to produce PHA in island environment and the PHA could be utilized to replace other plastic materials.

## Supplementary Information

Supplementary Information.

## Data Availability

The datasets generated and analysed during the current study are available in the OL757724 repository, link: https://www.ncbi.nlm.nih.gov/nuccore/OL757724.
