# Supplementary material for: Evaluation of polyhydroxyalkanoate (PHA) synthesis by Pichia sp. TSLS24 yeast isolated in Vietnam
Source: Sci Rep. 2023 Feb 23;13:3137. doi: 10.1038/s41598-023-28220-z (PMC9950484; doi:10.1038/s41598-023-28220-z)
Supplement: Supplementary file 1 — Supplementary Information. [file 41598_2023_28220_MOESM1_ESM.docx]

**<https://www.ncbi.nlm.nih.gov/nuccore/OL757724>**

**Pichia sp. strain TSLS24 5.8S ribosomal RNA gene, partial sequence; internal transcribed spacer 2, complete sequence; and large subunit ribosomal RNA gene, partial sequence**

GenBank: OL757724.1

[FASTA](https://www.ncbi.nlm.nih.gov/nuccore/OL757724.1?report=fasta) [Graphics](https://www.ncbi.nlm.nih.gov/nuccore/OL757724.1?report=graph)

[Go to:](https://www.ncbi.nlm.nih.gov/nuccore/OL757724#goto2161519457_0)

LOCUS OL757724 890 bp DNA linear PLN 13-DEC-2021

DEFINITION Pichia sp. strain TSLS24 5.8S ribosomal RNA gene, partial sequence;

internal transcribed spacer 2, complete sequence; and large subunit

ribosomal RNA gene, partial sequence.

ACCESSION OL757724

VERSION OL757724.1

KEYWORDS .

SOURCE Pichia sp.

ORGANISM [Pichia sp.](https://www.ncbi.nlm.nih.gov/Taxonomy/Browser/wwwtax.cgi?id=4925)

Eukaryota; Fungi; Dikarya; Ascomycota; Saccharomycotina;

Saccharomycetes; Saccharomycetales; Pichiaceae; Pichia.

REFERENCE 1 (bases 1 to 890)

AUTHORS Nguyen,T.T., Pham,C.K. and Bui,H.T.

TITLE Direct Submission

JOURNAL Submitted (08-DEC-2021) Biochemistry department, New Technology

Institute, 17 Hoang Sam, Nghia Do, Cau Giay, Ha Noi 0000, Viet Nam

COMMENT ##Assembly-Data-START##

Assembly Method :: Newbler v. 11/2021

Sequencing Technology :: Sanger dideoxy sequencing; Illumina

##Assembly-Data-END##

FEATURES Location/Qualifiers

source 1..890

/organism="Pichia sp."

/mol_type="genomic DNA"

/strain="TSLS24"

/db_xref="taxon:[4925](https://www.ncbi.nlm.nih.gov/Taxonomy/Browser/wwwtax.cgi?id=4925)"

[misc_RNA](https://www.ncbi.nlm.nih.gov/nuccore/OL757724.1?from=1&to=890) <1..>890

/note="contains 5.8S ribosomal RNA, internal transcribed

spacer 2, and large subunit ribosomal RNA"

ORIGIN

1 tctcttggtt ctcgcatcga tgaagagcgc agcgaaatgc gatacctagt gtgaattgca

61 gccatcgtga atcatcgagt tcttgaacgc acattgcgcc cctcggcatt ccggggggca

121 tgcctgtttg agcgtcgttt ccatcttgcg cgtgcgcaga gttgggggag cggagcggac

181 gacgtgtaaa gagcgtcgga gctgcgactc gcctgaaagg gagcgaagct ggccgagcga

241 actagacttt ttttcaggga cgcttggcgg ccgagagcga gtgttgcgag acaacaaaaa

301 gctcgacctc aaatcaggta ggaatacccg ctgaacttaa gcatatcaat aagcggagga

361 aaagaaacca acagggattg cctcagtagc ggcgagtgaa gcggcaagag ctcagatttg

421 aaatcgtgct ttgcggcacg agttgtagat tgcaggttgg agtctgtgtg gaaggcggtg

481 tccaagtccc ttggaacagg gcgcccagga gggtgagagc cccgtgggat gccggcggaa

541 gcagtgaggc ccttctgacg agtcgagttg tttgggaatg cagctccaag cgggtggtaa

601 attccatcta aggctaaata ctggcgagag accgatagcg aacaagtact gtgaaggaaa

661 gatgaaaagc actttgaaaa gagagtgaaa cagcacgtga aattgttgaa agggaagggt

721 attgcgcccg acatggggat tgcgcaccgc tgcctctcgt gggcggcgct ctgggctttc

781 cctgggccag catcggttct tgctgcagga gaaggggttc tggaacgtgg ctcttcggag

841 tgttatagcc agggccagat gctgcgtgcg gggaccgagg actgcggccg

//
